# Role of Liver X Receptor in AD Pathophysiology

**DOI:** 10.1371/journal.pone.0145467

**Published:** 2015-12-31

**Authors:** Adrián G. Sandoval-Hernández, Luna Buitrago, Herman Moreno, Gloria Patricia Cardona-Gómez, Gonzalo Arboleda

**Affiliations:** 1 Grupo de Muerte Celular, Instituto de Genética Universidad Nacional de Colombia, Bogotá, Colombia; 2 Departments of Neurology and Physiology/Pharmacology, The Robert F. Furchgott Center for Neural and Behavioral Science, SUNY Downstate Medical Center, Brooklyn, New York, United States of America; 3 Área de Neurobiología Celular y Molecular, Grupo de Neurociencias de Antioquia, Universidad de Antioquia, Calle 70, No. 52–21, Medellín, Colombia; Centre Hospitalier de l'Université Laval, CANADA

## Abstract

Alzheimer's disease (AD) is the major cause of dementia worldwide. The pharmacological activation of nuclear receptors (Liver X receptors: LXRs or Retinoid X receptors: RXR) has been shown to induce overexpression of the ATP-Binding Cassette A1 (ABCA1) and Apolipoprotein E (ApoE), changes that are associated with improvement in cognition and reduction of amyloid beta pathology in amyloidogenic AD mouse models (i.e. APP, PS1: 2tg-AD). Here we investigated whether treatment with a specific LXR agonist has a measurable impact on the cognitive impairment in an amyloid and Tau AD mouse model (3xTg-AD: 12-months-old; three months treatment). The data suggests that the LXR agonist GW3965 is associated with increased expression of ApoE and ABCA1 in the hippocampus and cerebral cortex without a detectable reduction of the amyloid load. We also report that most cells overexpressing ApoE (86±12%) are neurons localized in the granular cell layer of the hippocampus and entorhinal cortex. In the GW3965 treated 3xTg-AD mice we also observed reduction in astrogliosis and increased number of stem and proliferating cells in the subgranular zone of the dentate gyrus. Additionally, we show that GW3965 rescued hippocampus long term synaptic plasticity, which had been disrupted by oligomeric amyloid beta peptides. The effect of GW3965 on synaptic function was protein synthesis dependent. Our findings identify alternative functional/molecular mechanisms by which LXR agonists may exert their potential benefits as a therapeutic strategy against AD.

## Introduction

Alzheimer's disease (AD) is an age-dependent neurodegenerative disorder and a major public-health problem. The hallmark of the disease is the formation of two types of protein aggregates: extracellular amyloid plaques and intracellular neurofibrillary tangles (NFTs) which are composed mainly of hyper-phosphorylated tau protein, both of which are necessary for the definitive diagnosis of AD in humans [1]. The amyloid cascade hypothesis postulate that the disease begins with an accumulation of amyloid beta peptides (Aβ1–42 and Aβ1–40) that promotes the subsequent events—including neuroinflammation, kinase deregulation, tangles formation, neurite dystrophy, synaptic deficits—which ultimately leads to neuronal death and dementia [2]. The hippocampal formation, one of the brain areas involved in learning and memory, is the earliest affected structure in AD [3]. Within the hippocampus, the dentate gyrus (DG), has been described as one of the main areas where adult neurogenesis occurs and neurogenesis has been proposed to be affected in AD [4].

On the other hand, lipid metabolism has emerged as an important risk factor for AD, such that the patients with alterations in serum cholesterol levels have an increased risk of developing various neurodegenerative diseases including AD [5,6]. This observation is supported by studies reporting that patients who have undergone cholesterol lowering statin treatment have a lower prevalence of AD [7,8]. Genome wide association studies (GWAS) have also identified that polymorphisms in genes related with lipid metabolism associate with higher risk of AD [9], including the well-known allelic variants of ApoE [10] and ABCA7 [11].

Cholestrol within the central nervous system, serves many different functions. For instance, cholesterol depletion promotes neurodegeneration by inducing synaptic and dendritic dysfunction [12]. Liver X receptors (LXRα and LXRβ) belongs to the superfamily of nuclear receptors, act as cholesterol sensors through binding to their physiological ligands, most of which are oxygenated metabolites of cholesterol (i.e. oxysterols). LXRs can also be activated *in vivo* by additional agonists including glucose and glucose-6-phosphate [13]. However, the function of nuclear receptors in the brain during development and in adulthood, remains poorly understood [14]. Several studies have shown that LXRs are involved in dopaminergic neuron differentiation during the development of the ventral midbrain as well as in the increase of the number of dopaminergic neurons derived from embryonic stem cells [15]. LXRβ is more ubiquitously expressed and enriched in tissues of neuronal and endocrine origen than LXRα and it is expresed in neuronal cells [16], Knock-out mice of LXRα and/or LXRβ in a background of an amyloidogenic mouse model develop a more severe amyloid-related pathology [17].

Recently, it was reported that pharmacological activation of LXRs in murine models of AD partially reverted the cognitive impairment. This is thought to be mediated by the increased expression of ApoE and ABCA1 [18–21]. Nonetheless, the impact of such therapies on the amyloid load remains controversial. Previous studies using agonist that activated directly LXR (GW3965 and TO901317) or indirectly (Bexarotene) improved cognition using different paradigms; however there are discrepancies in the clearance of plaque load between reports. For instance GW3965 treatment in adult symptomatic Tg2765 mice produced increased expression of ApoE and ABCA1 and reduction in plaque load [18] while TO901317 treatment in APPSLxPS1 mice induced changes in molecular species of cholesterol without significant changes in plaque load [22]. The agonist of RXR (Bexarotene) that forms active heterodimers with LXRs or PPARs, caused rapid overexpression of ApoE and reduction in amyloid load after 3 to 7 days of treatment in 6-month-old APPswe/PS1Δe9 mice [23]. Other two groups using Bexarotene did not found changes in plaque load in the same model (7 and 11 months old) [24,25]

In order to identify new potential mechanisms that may play a role in the improvement of cognition in murine models of AD treated with LXRs agonists, we analyzed the effects of long-term LXR activation using the LXR agonist GW3965 administered for 12 weeks in 12-months-old 3xTg-AD mice. The 3xTg-AD mouse mimics more closely the human disease by developing both histological hallmarks of AD, amyloid and Tau pathologies with early synaptic dysfunction [25–27].

We observed that, in addition to improvement in cognitive function and increase in the expression of ApoE and ABCA1, ApoE showed a specific up-regulation in the treated 3xTg-AD mice localized mainly in neurons. There was also increased expression of neuronal stem cell markers such as nestin and the number of proliferating cells in the subgranular zone (SGZ). We did not find significant changes in the amyloid load. Our results agree with previous observations and postulate alternative novel mechanisms by which LXR agonists may exert their potential benefits as a therapeutic strategy against AD. Furthermore GW3965 prevented oligomeric Aβ 42 peptides induced long-term synaptic plasticity abnormalities in a protein synthesis dependent manner.

## Materials and Methods

### Ethical Statement

All animal procedures were performed in concordance with the Animal Research: Reporting *in Vivo* Experiments (ARRIVE) guidelines [28]. Also following the Guide for the Care and Use of Laboratory Animals, 8th edition, published by the National Institutes of Health (NIH) at SUNY-Downstate, and according to Colombian standards (law 84/1989 and resolution 8430/ 1993). These procedures were approved by Ethics Committee for Animal Experimentation of the University of Antioquia, Medellín, Colombia.

### Animals

Triple-transgenic AD mice (3xTgAD) were described in detail in [29] and wild-type littermates (WT) from the house colony of SPF (Species Pathogen Free) vivarium of SIU (Sede de Investigación Universitaria), at the University of Antioquia, Medellín-Colombia, were kept on a 12:12-h dark:light cycle and received food and water *ad libitum*. Special care was taken to minimize animal suffering and to reduce the number of animals used. A total of 24 3xTg-AD (12-month-old) and 17 WT (12-to 16-month-old) mice were used. Female and male animals were randomized and equally distributed into the groups. Animals were treated orally every day with 33 mg/kg GW3965 or with vehicle (Dimethyl sulfoxide: DMSO—dilution 20mg/ml) for 12 consecutive weeks, in groups of five animals in regular cages.

### Morris Water Maze

Hippocampal-dependent spatial memory was evaluated using a water maze paradigm [30]. The apparatus consisted in a white pool (100 cm in diameter and 54 cm high). The tank was filled with opaque water with non-toxic white gouache paint, kept at (22 ± 2°C) and a depth of 35 cm. The platform (10 cm of diameter) was located 1.5 cm below water surface during spatial learning and 1 cm above water surface during the visible session. Extra-maze visual cues around the room remained in a fixed position throughout the experiment.

Initial spatial training: the aim was to have the mice acquire a learning task with two daily sessions for five days, in which mice were trained to find the platform. Each session consisted of four successive trials and each trial began with the mice placed pseudo-randomly in one of four starting positions. Before the initial trial, animals were trained to stay for 30s on the platform. Forty-eight hours after the learning phase, animals were tested for retention during a 90s probe trial without the platform. During the probe trial, the latency to reach the exact platform location was determined. To control for differences between experimental groups in visual-motor abilities or motivation, latencies to reach the platform were evaluated with a visible platform (four trials) at the end of the retention task. Behavior was recorded by an automated system (Viewpoint, Lyon, France).

Spatial reversal training: after the initial spatial learning task, a reversal learning protocol was conducted with all animals. During the reversal learning, the hidden platform was moved to the nearest quadrant at right. The reversal learning was performed for two days, two trials per day similar to the initial training.

### Histology of Aβ plaques and neurofibrillary tangles

Mice were anesthetized with Ketamine/Xylazine and perfused trans-cardially with 4% paraformaldehyde in phosphate buffered saline (PBS), and the brains were removed and transferred into 30% sucrose solution. Brains were cut in 50 μm coronal sections with a vibratome (Leica VT 1000S, Leica, Nussloch, Germany) and stored at –20°C in anti-freezing medium until further processing. For diaminobenzidine staining, the sections were pretreated for 20 min in 0.1 M phosphate buffer (PB) (pH 7.4) with methanol (PB/methanol 1:1), at room temperature and 1% hydrogen peroxide to block endogenous peroxidase. Afterwards, sections were rinsed and incubated for 1 h in 0.1 M PB with 1% BSA, 0.3% Triton X-100. The slices were incubated with primary antibodies in the same buffer overnight at 4°C. Primary antibodies that recognize tau phosphorylated at S202/205 in mouse and human [31] (AT8,1:500; Thermo Scientific, Rockford, IL, USA), and 6E10 (SIG-39320; Signet/Covance, Dedham, MA) which recognizes human APP/Abeta. Subsequently, slices were incubated with biotinylated mouse secondary antibody and then incubated with ABC–HRP complex (Pierce Biotechnology; Rockford, IL, USA) for 2 h. Detection was developed with diaminobenzidine (DAB). Tissue was dehydrated and covered with mounting solution and observed under a Zoom-Microscope (Axio-Zoom-V16, Zeiss, Munich, Germany). Images were acquired with a Zeiss Axio Cam HRc digital color camera (1388x1040 pixels) using ZEN pro software (Zeiss, Munich, Germany). Semi-quantitative analysis of areas for each captured image was obtained by segmentation analysis using ImageJ software (NIH, USA). Areas identified as positive for immunoreactivity for 6E10 or AT8 after thresholding, were individually inspected to confirm the objects as plaques or hyperphosphorylated tau. The mean value of area was the averaged per treatment in each genotype group for the number of animals per group indicated in figure legends. Statistical analysis was done by ANOVA and Bonferroni post test, to establish comparisons between groups. Thioflavin-S (Thio-S) staining was assessed as previously described [32]. In short slides were rinsed consecutively in ethanol (70% then 80% for 1 minute respectively), subsequently incubated in Thio-S solution (1% in 80% of ethanol) for 15 min, and then rinsed consecutively in ethanol (80%, 70% for 1 minute). Finally, the slides were rinsed with distilled water twice and covered with glycerol jelly. Images were captured under fluorescence microscopy (20X) by using Confocal Microscopy (Nikon Eclipse C1 plus; Nikon Instruments Inc., Melville, NY, USA).

### Immunofluorescence

Brain coronal sections of 50 μm in anti-freezing medium were washed three times with PBS; auto-fluorescence was reduced with 50 mM NH_4_Cl for 10 min at room temperature. Tissue was permeabilized using 1% BSA with 0.3% Triton X-100 in 0.1 mM PBS for 1 hour and incubated with primary antibodies: anti-ApoE (mouse, 1:500; which was raised against a peptide sequence to recognize the polymorphic amino acid position 158 of Apo-E Abcam, Cambridge, MA, Cat#:AB1906), anti-nestin (1:50, #sc21249; Santa Cruz Biotechnology, Santa Cruz, CA, USA) which recognizes an epitope at the C-terminus of nestin of mouse origin., anti-Doublecortin (Rabbit, 1:250; Abcam Cambridge, MA, Cat#:AB18723) that recognizes residues within 300 to the C-terminus of Doublecortin, anti-GFAP (Rabbit, 1:500; polyclonal antibody obtained by the immunization with a preparation of full length human recombinant GFAP; Abcam Cambridge, MA, Cat#:AB7260), anti-NeuN (Rabbit, 1:500; policlonal antibody obtained by immunization with a recombinant fragment corresponding to Human NeuN aa 1–100 (N terminal); Abcam Cambridge, MA, Cat#:AB104225), anti-Histone H3 (Rabbit, 1:1000; polyclonal antibody obtained by immunization with a synthesized phosphopeptide derived from human Histone H3 around the phosphorylation site of serine 10; Abcam Cambridge, MA, Cat#:AB47297), After overnight incubation at 4°C, fluorescent-labeled secondary antibodies were used: Alexafluor 488 (1:2500; Invitrogen, Grand Island, NY), Alexafluor 568 (1:2500; Invitrogen, Grand Island, NY), Goat Anti-Rabbit IgG H&L (AMCA) (1:500; Abcam Cambridge, MA, Cat#:ab123435). Nuclei were stained with Hoescht 33258 (Invitrogen H-1398, Grand Island, NY).

### Confocal analysis, colocalization and counting

A Nikon Eclipse C1 confocal microscopy plus (Nikon Instruments Inc., Melville, NY, USA) was used to determine the protein expression and colocalization. The slices were selected always at the same ventro-dorsal location and images were captured under 20X magnification. Two consecutive sections from each mouse (4 mice total per treatment) for the different regions of the hippocampus were analyzed using ImageJ software (National Institutes of Health, Bethesda, MD). For colocalization of immunohistochemical markers Argon 488, Helium Neon 543 lasers, 408 diode and 60X/1.4 oil lens were used, the images where analyzed using the colocalization threshold program included in the Fiji software package (http://fiji.sc/wiki/index.php/Fiji). To determine the percentage of neurons of the cell population with increased ApoE, we used captured images of 20X magnification (Figures A and B in S2 File.). We then conducted a manual counting of the number of cells with increased ApoE and the number of cells colabeled with NeuN in the granular layer of DG. Values are presented as mean ± S.E.M. The same method was used to analyze the number of proliferative cells in the subgranular zone (SGZ) of the hippocampus.

### Western Blot and Elisa

Following the pharmacological treatment, all animals received behavioral training, and then the brains were extracted and dissected manually. Tissues (cerebral cortex and hippocampus) were homogenized using 300 μl of ice-cold buffer RIPA (Pierce Biotechnology; Rockford, IL, USA) containing complete protease inhibitor cocktail tablets and phosphatase inhibitor cocktail tablets (Complete, PhosSTOP, Roche Applied Science, Mannheim Germany) in a homogenizer (PRO Scientific, Oxford, Connecticut, USA) at full speed for 20 s. The homogenate was sonicated at 30% output for 10 s, then centrifuged (12,500 g) at 4°C for 45 min in a microcentrifuge (Thermo Scientific, Rockford, IL, USA). The pellet (RIPA insoluble fraction) was resuspended in an equal amount of 5 M guanidine hydrochloride in 50 mM Tris-HCl, pH 8.0 for 3 h to evaluate fibrillar Aβ. Protein concentration of supernatant fractions was determined using the BCA protein assay kit (Thermo Scientific, Rockford, IL, USA) and twenty microgram of protein samples were run in polyacrylamide gel at 100 V. After electrophoresis, proteins were transferred to a PVDF membrane (Amersham Hybond™-P; GE Healthcare, Buckinghamshire, UK) and incubated in blocking buffer (5% powder milk in TBS-Tween 20) for 1 h at 48°C until probed. The PVDF membrane was then incubated with 5 ml of monoclonal primary antibodies [anti-ApoE (mouse, 1:1000, Abcam, Cambridge, MA, Cat#:AB1906), Anti-ABCA1(mouse, 1:1000, Abcam, Cambridge, MA, Cat#:AB66217)] in blocking buffer for 2 h at room temperature or overnight at 4°C, washed 3 times in TBS-Tween (5 min/wash), followed by incubation with peroxidase conjugated secondary antibody for 1 h (1:2000) and washed 3 times with TBS-Tween. Bound antibody was detected using the ECL system (Thermo Scientific, Rockford, IL, USA). ELISA for Amyloid Aβ (x-40) and Amyloid Aβ (x-42) was done by using RIPA soluble Aβ42 fraction using a commercial kit (Covance, SIG-38954, SIG-38956, Princeton, NJ, USA) following the manufacturer’s instructions.

Electrophysiology: Hippocampal horizontal slices were obtained as previously described [33]. In short, mice were anesthetized, brains removed and placed in modified artificial cerebral spinal fluid (aCSF) bubbled with O_2_ and CO_2_ (pH 7.4) and sectioned through the ventral hippocampus into 400 μm-thick slices. Field extracellular potentials were recorded in the CA1 stratum radiatum with glass electrodes filled with NaCl 150 mM (2–3 MΩ resistance) which were elicited by stimulating the Schaeffer collateral fibers using a tungsten bipolar electrode. Input-ouput relationship curves were obtained and a stimulus evoking ~40% of the maximum response was selected for the rest of the experiment. For the long-term potentiation (LTP) experiments a stimulus as described above was used, a baseline of test responses was obtained (15 min with an inter-stimulus interval of 30 s), and a high frequency stimulation (tetanus: one train of 100 stimuli at 100 Hz) was used to induce synaptic LTP. Responses were recorded for 90 min after tetanization. The field-EPSP (fEPSP) slope was measured and expressed as percentage of baseline. The results are expressed as mean ± S.E.M.

The direct effect of GW3965 and/or anisomycin 25 μM on the same synaptic response were evaluated every 30 s after obtaining a baseline, for additional 90 min. Oligomeric Aβ42 were prepared as described before [34]. Aβ42 and scrambled-Aβ42 peptides were obtained from American Peptide. Lyophilized Aβ peptides were resuspended in 1,1,1,3,3,3-hexafluoro-2-propanol (HFIP; Fluka); after drying, the pellets were diluted in DMSO (Sigma). Aβ/DMSO solutions were re-suspended in PBS buffer to the desired concentration 12 h before use. This peptide protocol induces oligomerization of Aβ peptides. Western-blot analysis using 6E10 antibodies showed that the main bands corresponded to dimers and trimers as shown previously [34]. Slices were incubated with 200 nM Aβ42, or scrambled-Aβ42 for 40 min and then high frequency stimulation was delivered, with or without other drugs as indicated in figure legends.

## Results

### GW3965 treatment rescues cognitive deficits in 3xTg-AD mice

**Fig 1:**
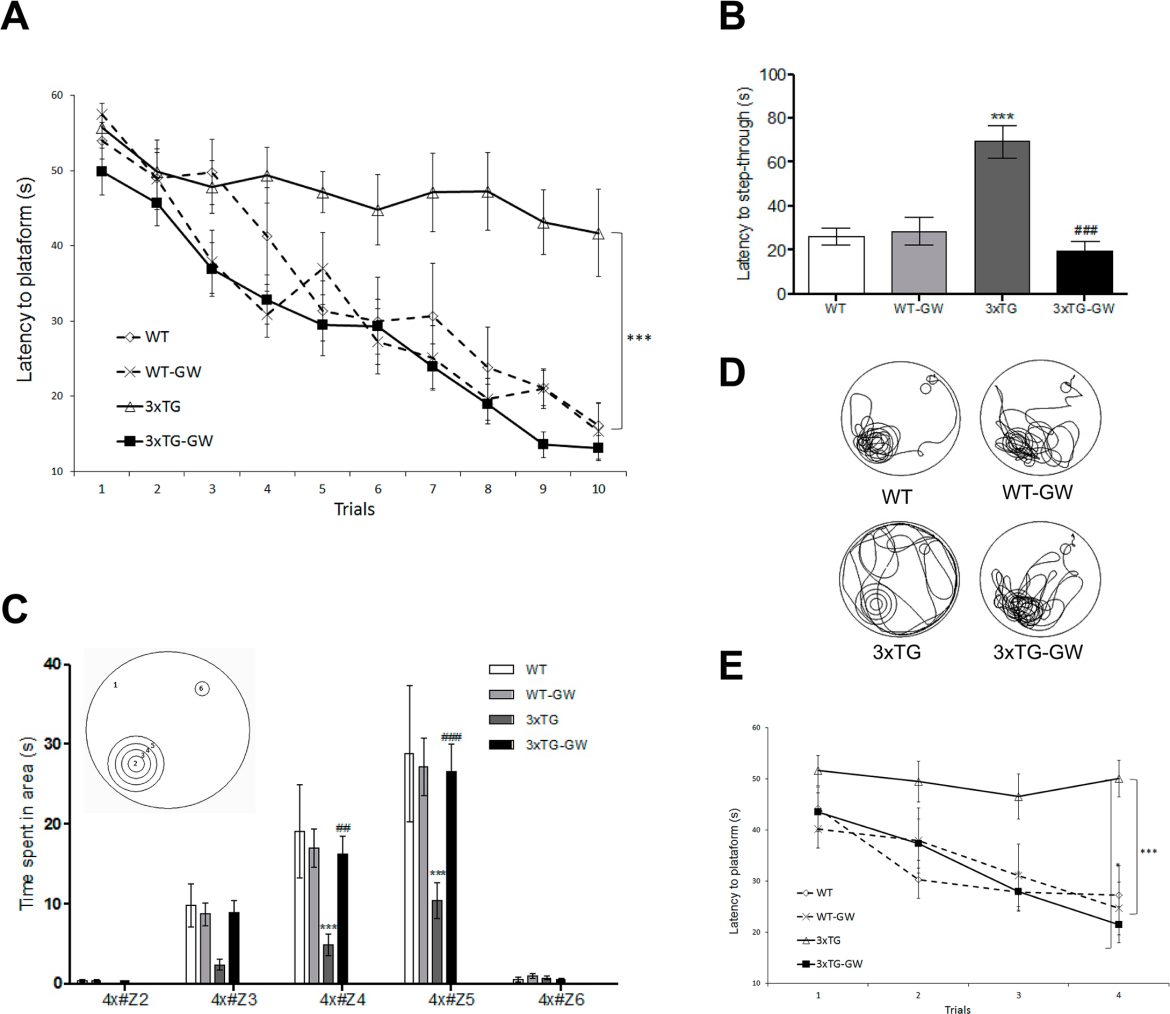
LXR agonist restores memory and cognition in the 3xTg-AD mice. Spatial learning and memory were evaluated by means of the MWM after 12 weeks of treatment with the LXR agonist GW3965. **(A) Learning task:** treated 3xTg-AD mice took significantly less time to learn the location of the hidden platform. Statistical significant differences were found by using two-way ANOVA. *** p<0.001. **(B-D) Retention tasks: (B)** latency to step-through place of platform in seconds. Significant differences were found by using ANOVA followed by Tukey's multiple comparison test; **(C)** 4X analysis: time spent nearest the platform; **(D)** Representative samples of paths taken during the retention task illustrate the marked preference between treated and untreated WT and 3xTg-AD mice. **(E) Reversal learning:** treated 3xTg-AD mice took significantly less time to learn the new location of the hidden platform after two days of trials. All data were expressed as mean ± S.E.M. *** represents p < 0.001 compared with WT; ^###^ represents p < 0.001 compared with untreated 3xTg-AD. Cohort sizes were: WT, n = 7; WT treated, n = 10; 3xTg-AD untreated, n = 8; treated 3xTG, (Randomized Females and Males) n = 12. Learning task: all data were expressed as mean ± S.E.M. *** represents p < 0.001, compared with WT; ^###^ represents p < 0.001 compared with untreated 3xTg-AD.

We initially examined the effects in cognition by using the Morris Water Maze (WMW) task to determine if a long term LXR-activation contributes to improvement of spatial learning and memory in 3xTg-AD mice. In the learning task (Fig 1A), untreated WT mice performed significantly better than untreated 3xTg-AD mice on 5 days of trials (p<0.001). When 3xTg-AD mice were treated with GW3965, there was no significant difference with WT in these tasks. No significant differences were observed between untreated and treated WT. In retention task (Fig 1B), untreated WT had significantly less time of latency to step through the platform than untreated 3xTg-AD mice (p<0.001); GW3965-treated 3xTg-AD mice had no significant differences with WT. No statistically significant differences in latency were observed between untreated and treated WT The retention task analyzed in a 4X proximity design showed that treated 3xTg-AD mice—compared with untreated 3xTg-AD mice—spent more time swimming near the platform (p<0.001). In addition, no significant differences were found between treated and untreated WT (Fig 1C and 1D). Spatial reversal training was also assessed and the results were similar to those obtained for learning task. Untreated WT performed significantly better than untreated 3xTg-AD mice on 2 days of trials and untreated 3xtg-AD mice did not show any significant decrease in latency of escape over 2 days of trials. When 3xTg-AD mice were treated with GW3965, there was no significant difference with WT in this task. No differences were observed between untreated and treated WT mice in any of the measures (Fig 1E). These data indicate that GW3965 improves the hippocampal-dependent behavior tested by MWM in 3xTg-AD and does not significantly affect performance in WT mice.

### GW3965 treatment does not modify the pathological hallmarks of AD

**Fig 2:**
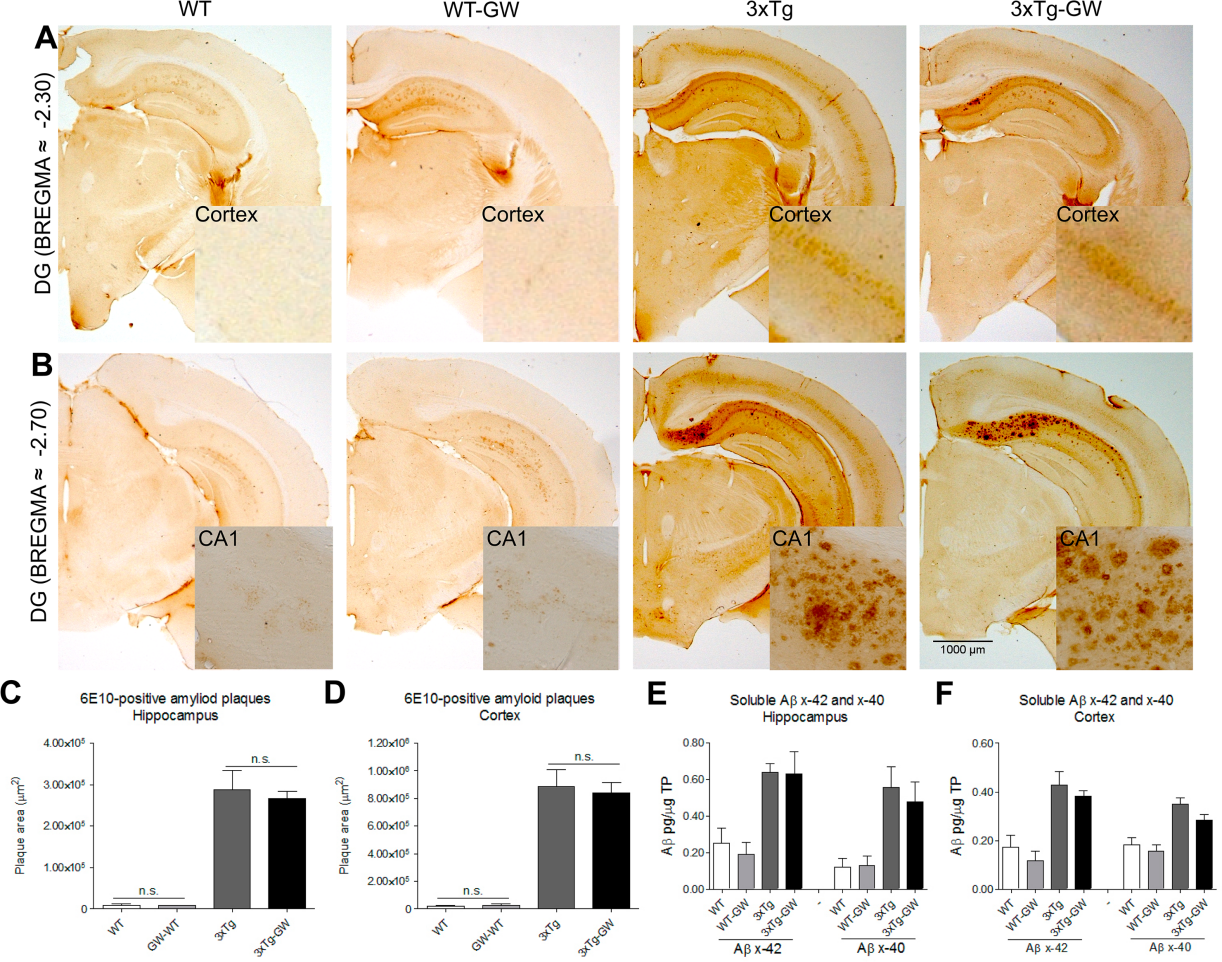
GW3965 treatment did not produce detectable reduction in amyloid beta load. Amyloid plaque deposition in brains of treated and untreated mice groups were evaluated by immunohistochemistry and soluble Aβ (1–42) by ELISA. **(A-B)** Representative micrographs of brain sections of Aβ immunohistochemistry with antibody anti-Aβ (6E10). **(C-D)** No significant differences were observed on the anti-Aβ positive area in the hippocampus or cortex of treated 3xTg-AD mice. Data were expressed as mean ± S.E.M. Statistical analysis was done by one way ANOVA followed by Tukey's multiple comparison test. Females n = 4 per 3xTg-AD group. **(E-F)** RIPA soluble Aβ42 and Aβ40 in the hippocampus or cortex were measured by ELISA. Quantification data were expressed as mean ± S.E.M. Statistical analysis was done by one way ANOVA followed by Tukey's multiple comparison test. n = 5 per group.

**Fig 3:**
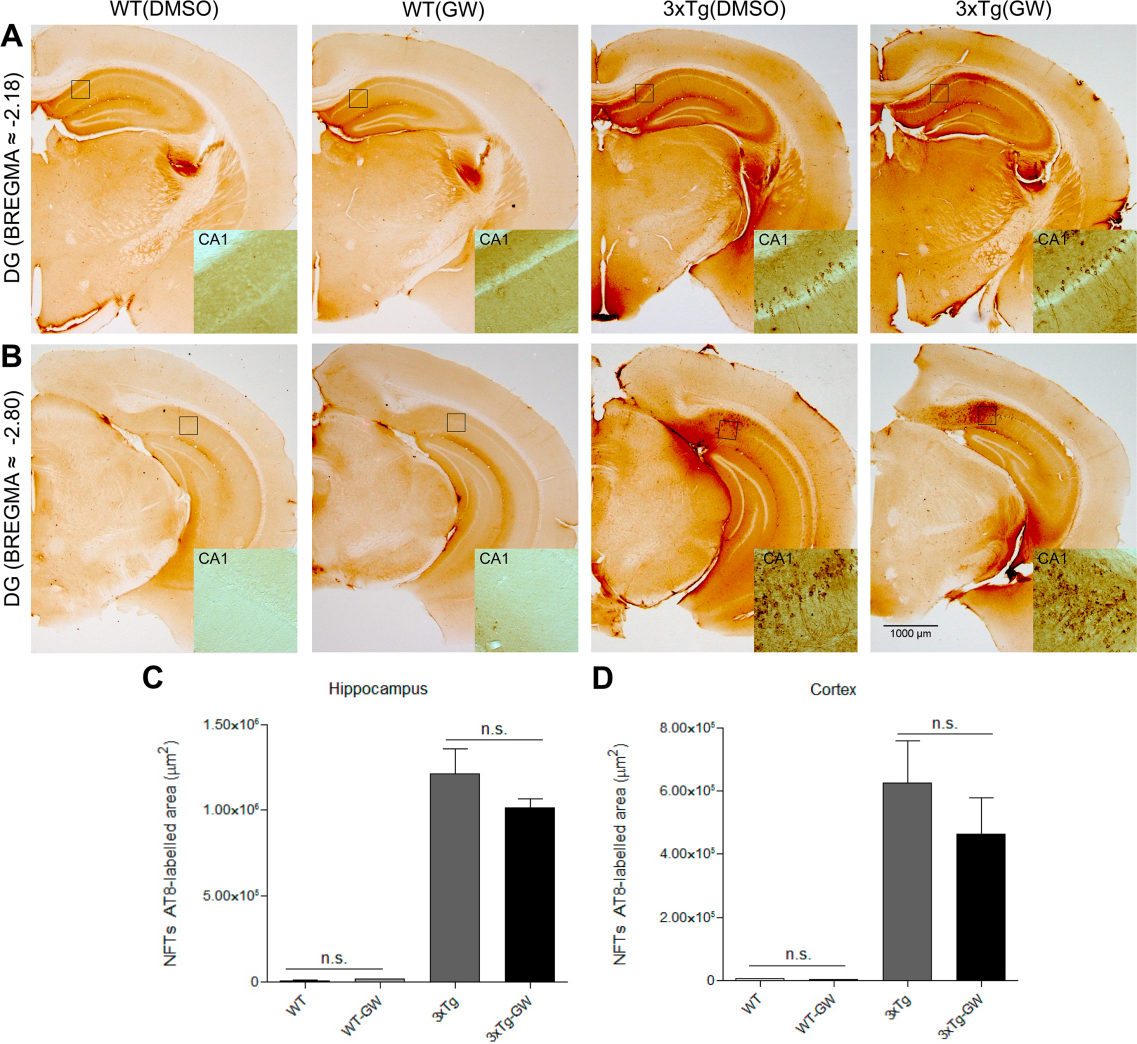
Tau pathology is not significantly reduced by LXR activation. **(A-B)** Presence of aggregated phospho-tau in brains of treated and untreated mice groups was evaluated by immunohistochemistry with anti-phospho-tau antibody (AT-8). **(C-D)** No significant changes were found in the quantification of aggregated phospho-tau occupied area within the hippocampus or cortex in treated mice. Data were expressed as mean ± S.E.M. Statistical analysis was done by one way ANOVA followed by Tukey's multiple comparison test. n = 4 Females per 3xTg-AD group.

To assess whether LXR-activation induced changes in amyloid plaque load and phosphorylated-Tau and if these changes were associated with the cognitive improvement observed, we performed immunohistochemistry analysis by using DAB detection and Thio-S staining. No significant differences in the amyloid plaque load or soluble Aβ (RIPA soluble Aβ (1–40) and RIPA soluble Aβ (1–42) were observed (Fig 2); neither changes were observed in aggregated phospho-tau (Fig 3A and 3B). Thio-S staining analysis to evaluate protein aggregation, showed no significant difference between treated and untreated animals (Figure A and Figure B in S1 File).

### GW3965 treatment increases the expression level of ApoE and ABCA1 proteins

**Fig 4:**
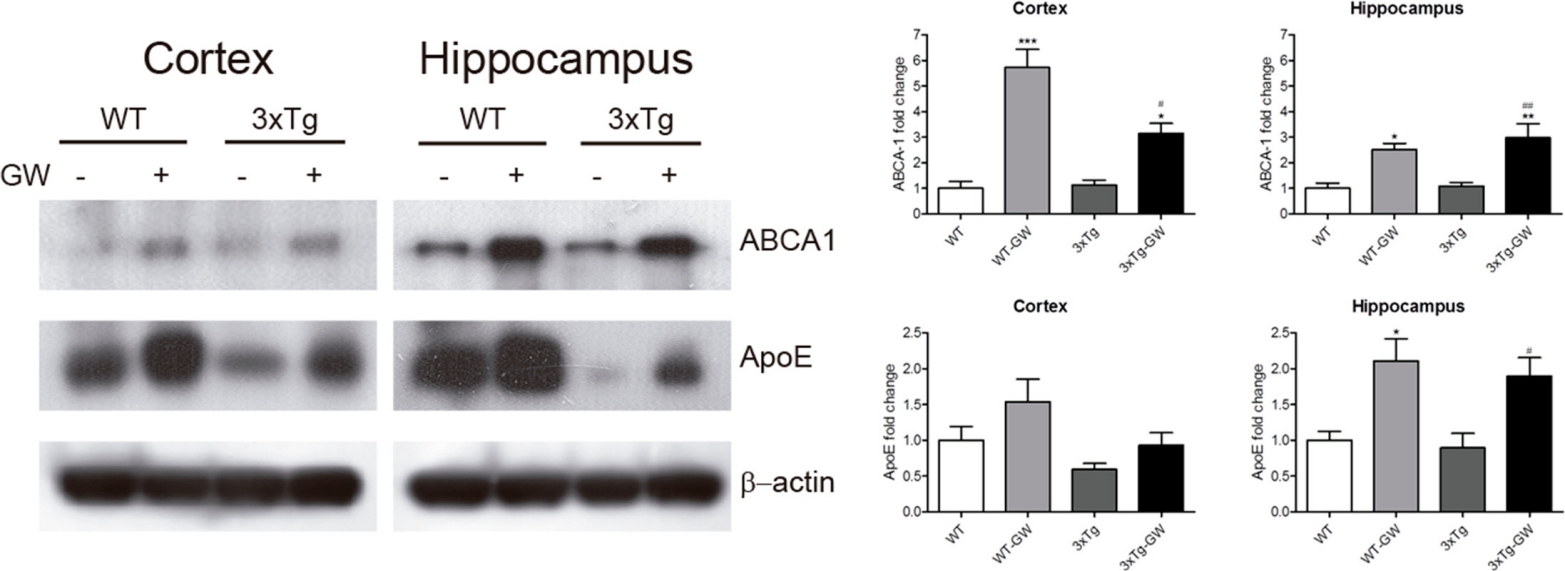
Brain ApoE and ABCA1 protein levels were up regulated by LXR agonist treatment. Brain levels of LXR target genes were evaluated in the hippocampus and cortex by western blot analysis. **(A)** Representative images of ApoE, ABCA1 and β-actin Western Blot detection in cortex and hippocampus in WT or 3xTg-AD mice treated with either vehicle or GW3965. **(B)** Densitometric quantification of Western blot assay of ApoE and ABCA1 in brain lysates of cortex and hippocampus from WT or 3xTg-AD mice treated with either vehicle or GW3965. Samples from each mouse were normalized to actin and expressed as fold-change. Data is expressed as mean ± S.E.M. Statistical analysis was done by one way ANOVA with Tukey's multiple comparison test, n = 5 per group. ***: p < 0.001; *: p < 0.05 compared with WT; ^#^: p < 0.05; ^##^: p < 0.01 compared with untreated 3Tg-AD.

To provide an overall view of ApoE and ABCA1 expression, we performed a western blot (WB) analysis of hippocampal and cortical tissue of various groups of animals (wild type and 3xTg-AD animals, untreated or treated with GW3965) (Fig 4).

In cerebral cortex, ABCA1 expression increased 6-fold in treated WT compared with untreated WT (n = 5 per group, p<0.001); there was a 3-fold increase in ABCA1 expression in treated 3xTg-AD compared with untreated WT and untreated 3xTg-AD animals (n = 5 per group, p<0.05) (Fig 4B). In the hippocampus, ABCA1 expression increased 3-fold in treated WT compared with untreated WT (n = 5 treated and untreated, p<0.05); there was a 3-fold increase in ABCA1 in treated 3xTg-AD compared with untreated WT and untreated 3xTg-AD animals (n = 5 per group, p<0.01) (Fig 4B).

ApoE expression was increased 2-fold in the hippocampus of treated WT mice compared with untreated WT animals (n = 5 treated and untreated, p<0.05). A 2-fold increase in ApoE expression was also observed in the treated 3xTg-AD group compared with untreated WT and untreated 3xTg-AD groups was also observed (n = 5 per group, p<0.05) (Fig 4B). No significant changes were observed in ApoE expression in the cerebral cortex (Fig 4B).

### ApoE expression in sub-regions of the hippocampus and cortex

**Fig 5:**
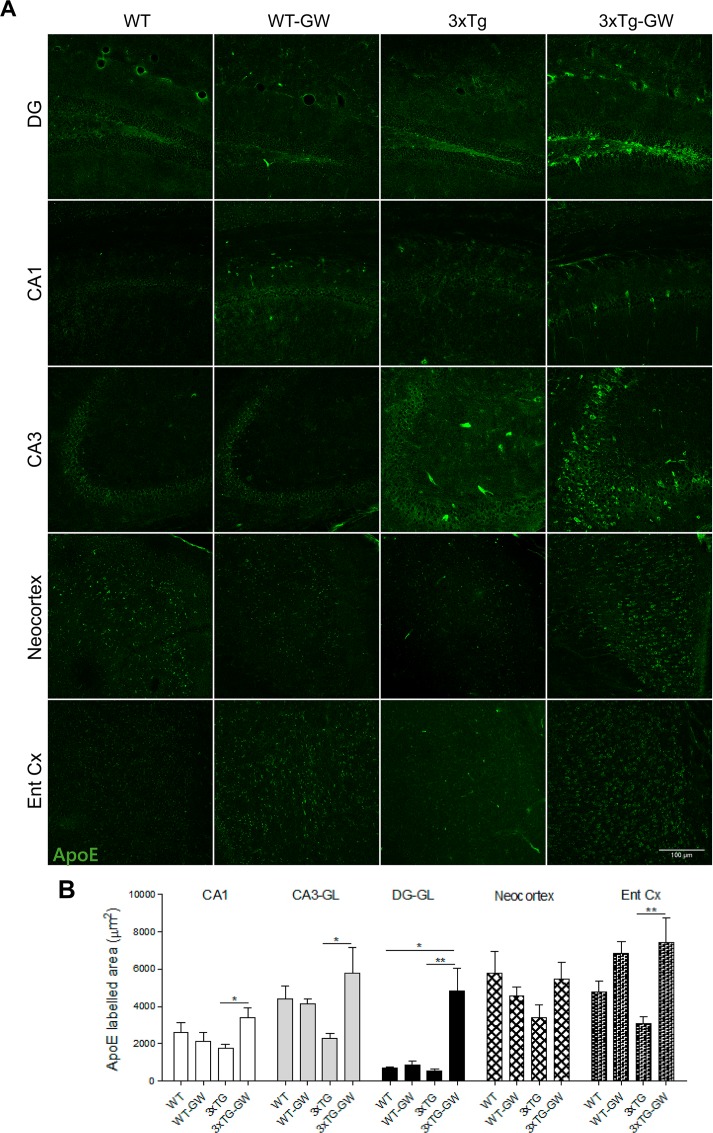
LXR agonist increased ApoE immunofluorescence particularly on 3xTg-AD mice. **(A)** Representative micrographs of ApoE immunofluorescence (Green) using confocal microscopy in the different analysed regions: Hippocampus (DG, CA3 and CA1) Entorhinal cortex (Ent Cx) and Neocortex in treated, untreated WT mice and 3xTg-AD mice. (B) Comparative analysis between treatment groups of the immunoreactive area occupied by ApoE, the data is expressed as mean ± S.E.M. Statistical analysis was performed by one-way ANOVA followed by Bonferroni post test.*: p<0.05, **: p<0.01. n = 4 per group.

To determine the specific brain regions in which ApoE changes were observed in treated 3xTg-AD mice, we analyzed its expression in the CA1, CA3, and DG regions of the hippocampus, in the neocortex (retrosplenial agranular and granular cortex (RSA/RSG Cortex) and entorhinal cortex by confocal microscopy and WB. Examination of different regions revealed no significant changes between areas of immunoreactivity of treated compared with untreated WT (Fig 5B). However, a significant 2-fold signal intensity increase in ApoE expression was found in entorhinal cortex for treated 3xTg-AD compared with untreated 3xTg-AD mice (Fig 5A and 5B). Similar analysis of the CA1 and CA3 areas of the hippocampus in 3xTg-AD animals revealed a 2-fold increase in treated compared with untreated animals (Fig 5A and 5B) Of note, the highest increase (5-fold) in ApoE expression was observed in the granular cell layer (GCL) of DG in the hippocampus of treated 3xTg-AD compared with untreated 3xTg-AD animals (P<0.01) (Fig 5B). The ApoE expression increase in treated 3xTg-AD mice was also significantly higher than that in treated and untreated WT (p<0.05, controlling for multiple comparisons using Tukey´s test) (Fig 5A and 5B).

### Increased ApoE expression induced by GW3965 in 3xTg-AD is mainly localized in NeuN positive cells of the Dentate Gyrus

In the normal adult brain, ApoE is expressed predominantly in a particular subpopulation of astrocytes; however, different reports indicate that ApoE is also expressed by neurons following brain injury [35]. In order to define the cell population responsible for the observed increased expression of ApoE in the treated 3xTg-AD mice, we evaluated the immunoreactivity of glial fibrillary acidic protein (GFAP) and NeuN and its colocalization with ApoE.

**Fig 6:**
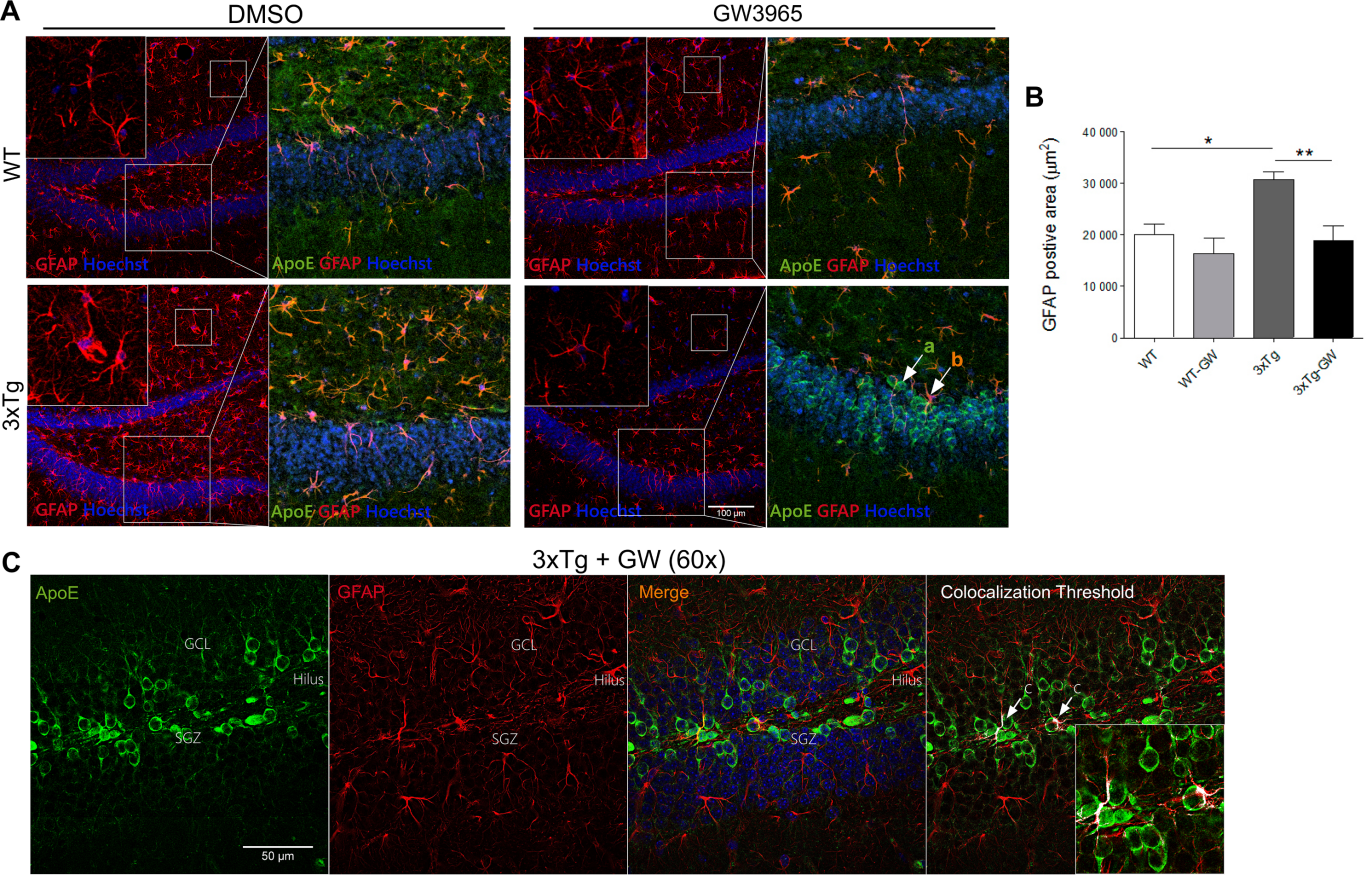
LXR activation reduces astrogliosis in treated 3xTg-AD. **(A)** Representative microphotographs of GFAP (Red) immunohistochemistry and Hoechst and caption of granular layer showing ApoE (Green), GFAP (Red) and Hoechst (Blue) using confocal microscopy in the DG of hippocampal slices. Shown are: in the left upper panel WT mock (DMSO) treated slice showing baseline immunoreactivity. Right upper panel slice treated with GW, demonstrating no significant changes upon treatment. Lower panels are 3xTg-AD mice mock and GW treated slices, indicating a significant reduction of GFAP immunoreactivity upon GW treatment. Two different populations of cells are identified: (a) cells expressing ApoE and (b) cells expressing ApoE and GFAP. Also shown are close up views of molecular layer regions, which are included in the right upper corner of all images, to appreciate the change in morphology of astrocytes in 3xTg-AD treated with GW3965. **(B)** Quantitative analysis of the data presented in A, indicating the significant changes in GFAP positive areas produce by GW3965 in treated 3xTg-AD. Note that 3xTg-AD mice have a significant increased GFAP area, indicating astrogliosis, which is at baseline level after GW treatment. Statistical analysis was performed by one-way ANOVA followed by Bonferroni post test. **(C)** Representative close-up views (60X) micrographs of GFAP (Red) and ApoE (Green) immunofluorescence and Hoechst (Blue) of DG granular layer of treated 3xTg-AD mice under confocal microscopy. The colocalization threshold indicated in white showed a minimal population of cells with increased ApoE expression. Data are expressed as mean ± S.E.M. Differences are against untreated WT: *: p<0.05, **: p<0.01; n = 4 per group.

First, we found that 3xTg-AD mice present increased gliosis compared with WT mice, shown by the larger area of positive GFAP immunofluorescence (Fig 6A and 6B), consistent with previous reports [36]. Importantly, LXR activation decreased the area of GFAP immunoreactivity in 3xTg-AD mice to the level of control WT mice (Fig 6A and 6B). These results are in line with the reported anti-inflammatory effect of LXR receptors [17].

**Fig 7:**
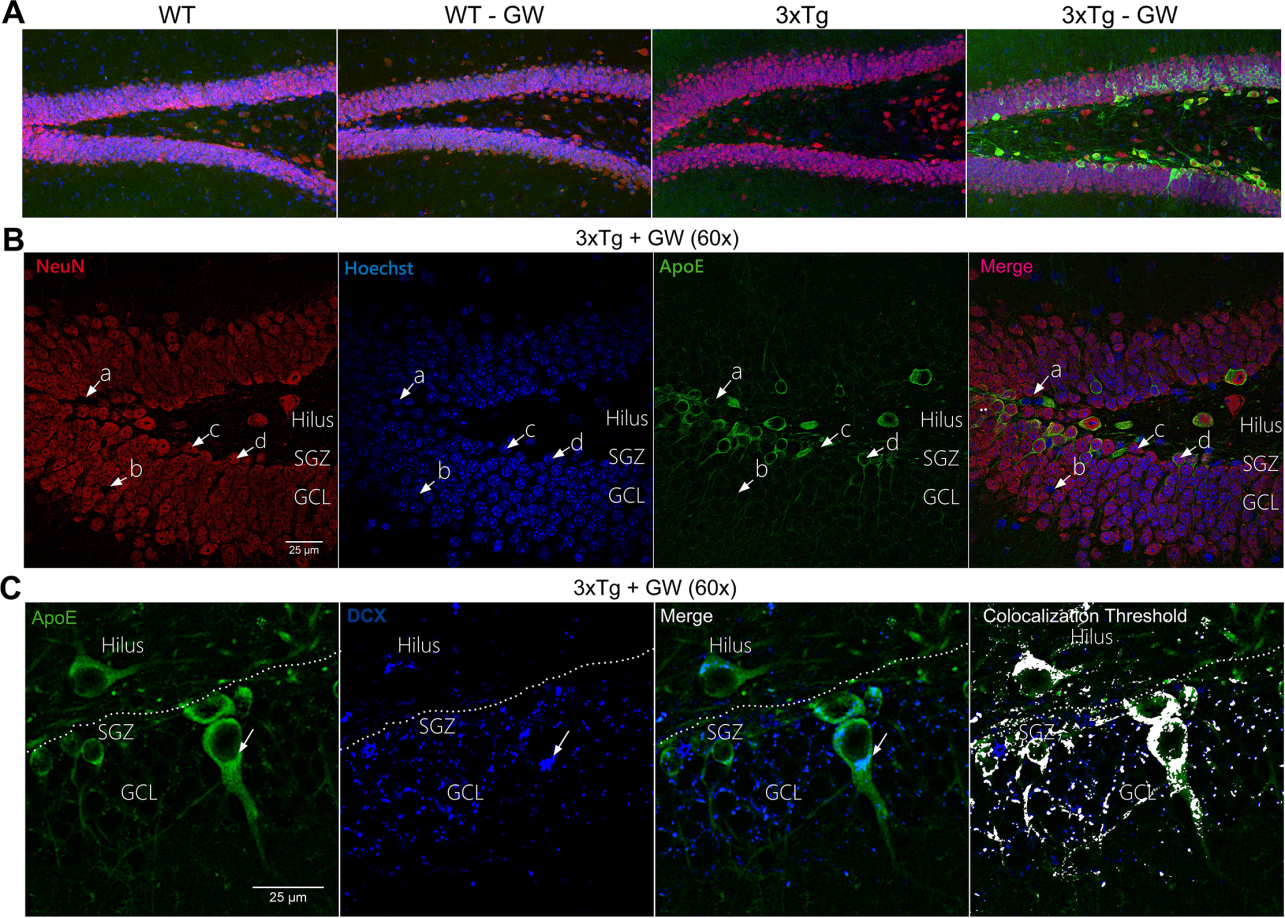
Neuronal localization of up-regulated ApoE in treated 3xTg-AD mice. **(A)** Representative micrographs of DG showing the immunofluorescence of ApoE (Green), NeuN (Red) and Hoechst (Blue) of treated and untreated WT and 3xTg-AD mice using confocal microscopy (20X). **(B)** Representative micrographs of GL of DG of treated 3xTg-AD mice showing immunofluorescence of ApoE (Green), NeuN (Red) and Hoechst (Blue) at 60X magnification **(C)**) Representative micrographs of GL of DG of treated 3xTg-AD mice showing immunofluorescence of ApoE and Doublecortin (DCX) (Blue) at 60X magnification. n = 4 per group.

Second, in order to identify the cell populations that showed increased expression of ApoE, we performed co-immunofluorescence analysis of ApoE, NeuN and Hoechst to label neurons, and ApoE, GFAP and Hoechst to identify astrocytes. In the first staining using NeuN, four population of cells were identified; (Fig 7B, a) cells expressing high levels of ApoE; (Fig 7B, b) cells with marked nuclei only (positive Hoechst); (Fig 7B, c) NeuN positive cells colocalized with Hoechst; and (Fig 7B, d) NeuN-positive cells colocalized with Hoechst in ApoE positive cells. The latter represented the largest population of cells with overexpression of ApoE (86±12%) (Fig 7, Figure A, Figure B, Figure C and Figure-D in S3 File). The astrocytes analysis using anti-GFAP showed that all GFAP-positive cells expressed ApoE in the DG molecular layer (ML). The expression pattern was similar to that reported in previous studies [35] (Fig 6A, b) and we also found a new population of cells in GL expressing only ApoE (Fig 6A, a). The relative fluorescence intensity (RFI) analysis of ApoE in the DG granular layer (GL) and ML showed a significant increase of ApoE signal in the GL of GW3965 treated 3xTg-AD mice (S2 File). The colocalization analysis of ApoE and GFAP in the GW3965 treated 3xTg-AD mice in the GL of DG, showed that the major expression of ApoE did not colocalize with GFAP positive cells, and also the colabeled cells for both GFAP and ApoE were mainly found in the SGZ (subgranular zone) as indicated by the threshold colocalization analysis (Fig 6C).

Since overexpressed ApoE is mainly localized in neurons, we evaluated the thickness of NeuN positive immunoreactivity in the GCL of DG as an indirect measure of the number of mature neurons, in order to determine if ApoE induces neuronal maturation. However, we observe no significant changes (Figure E and Figure F in S3 File).

### GW3965-induced increased expression of neuronal stem cells (NSC) and proliferation markers in dentate gyrus

**Fig 8:**
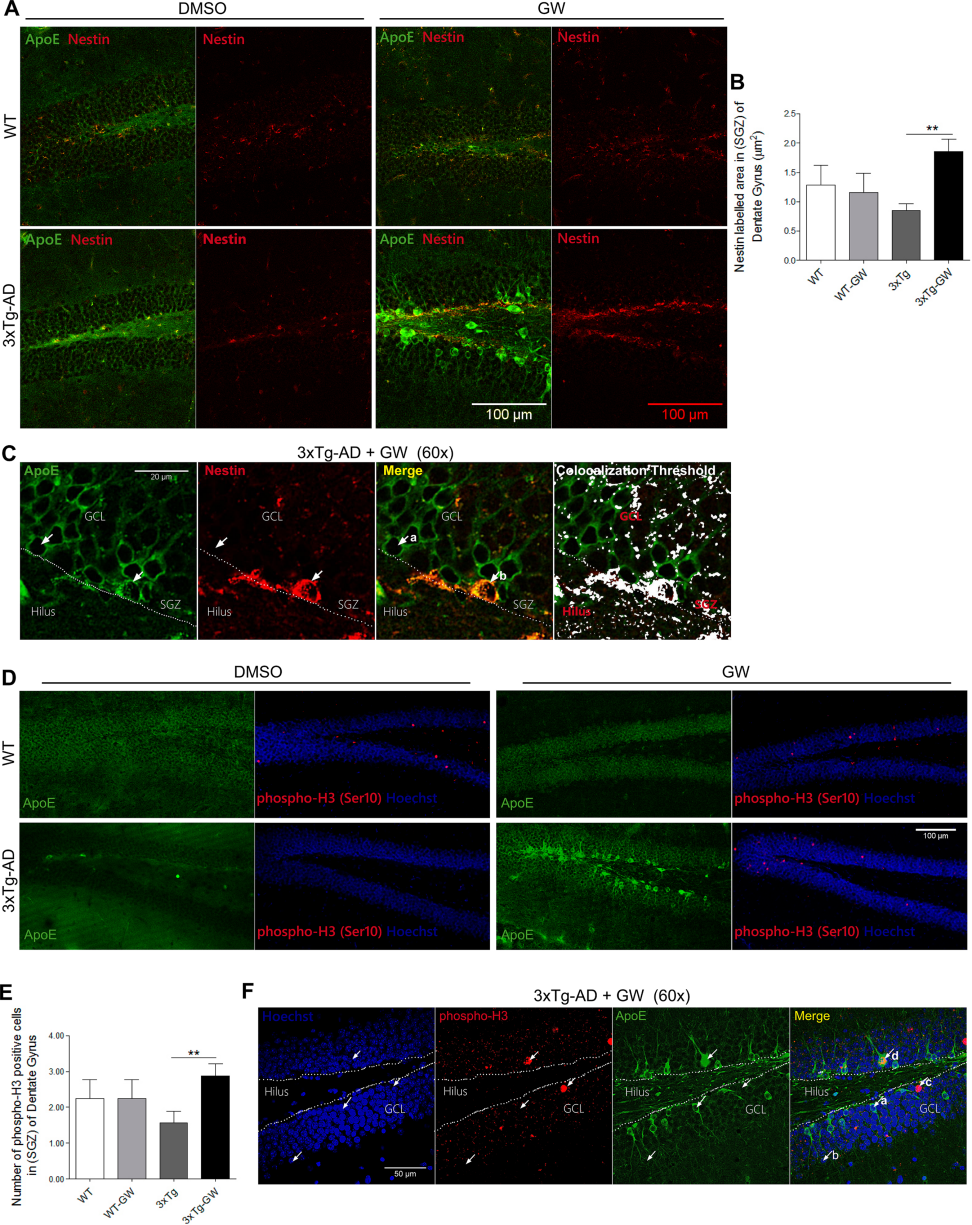
GW3965 increases the nestin immunoreactivity and the proliferating cells number in hippocampus of 3xTg-AD mice. **(A)** Representative micrographs of multiple immunofluorescence of ApoE (Green) plus nestin (Red) and side by side only nestin (Red) immunofluorescence in DG using confocal microscopy for treated and untreated WT and 3xTg-AD mice. **(B)** Quantification of nestin positive immunofluorescence in the SGZ of DG. **(C)** Representative micrographs of ApoE (Green) and nestin (Red) immunofluorescence (60X), the colocalization threshold of ApoE and nestin indicates that there is a population of cells with increased ApoE expression. **(D)** Representative micrographs of DG of treated and untreated WT and 3xTg-AD mice showing the immunofluorescence of ApoE and side by side phospho-Histone H3 (Ser10) (Red) plus Hoechst (Blue). **(E)** Quantification of the number of positive-nuclei labeled with pHH3 (Red) and Hoechst (Blue). **(F)** Representative micrographs of ApoE (Green) and phospho-H3 (Red) immunofluorescence and nuclei Hoechst (Blue,60X), showing that there are proliferating cells with increased ApoE expression. Data are expressed as mean ± S.E.M. Statistical analysis was performed by one-way ANOVA followed by Bonferroni post test: *:p<0.05; **: p<0.01, n = 4 per group.

Adult neurogenesis is dynamically regulated by different stimuli. Under normal conditions, neurogenesis is restricted to two specific brain regions, including the SGZ of DG, where new dentate granular cells are generated. We observed that the LXR agonist treatment increased ApoE protein expression mainly in neurons of the GCL of DG particularly in the 3xTg-AD mice; however, there was a small population of ApoE overexpressing cells that were not neurons (Fig 7B, a) and expressed GFAP (Fig 6C, a). This observation may indicate a different population of cells, most probably NSC. In order to answer this question, we assessed the coimmunoreactivity of nestin (a NSC marker) with ApoE in the SGZ. LXR agonist treatment of 3xTg-AD mice increased the area of nestin staining as compared with untreated 3xTg-AD animals by 2 folds (P = 0.01). However, no significant changes were observed between treated and untreated WT mice (Fig 8A and 8B). To determine if the population of NSC analyzed are functional and proliferate, we assessed the number of phospho-Histone H3 (Ser10) (pHH3)-positive nuclei. There was a significant increase in proliferative cells (+pHH3 cells) in treated 3xTg-AD mice compared with untreated 3xTg-AD mice, and no significant changes were observed in treated compared with untreated WT mice (Fig 8D and 8E).

### GW3965 rescues amyloid beta induced-LTP abnormalities in hippocampal slices, in a protein-synthesis-dependent manner

**Fig 9:**
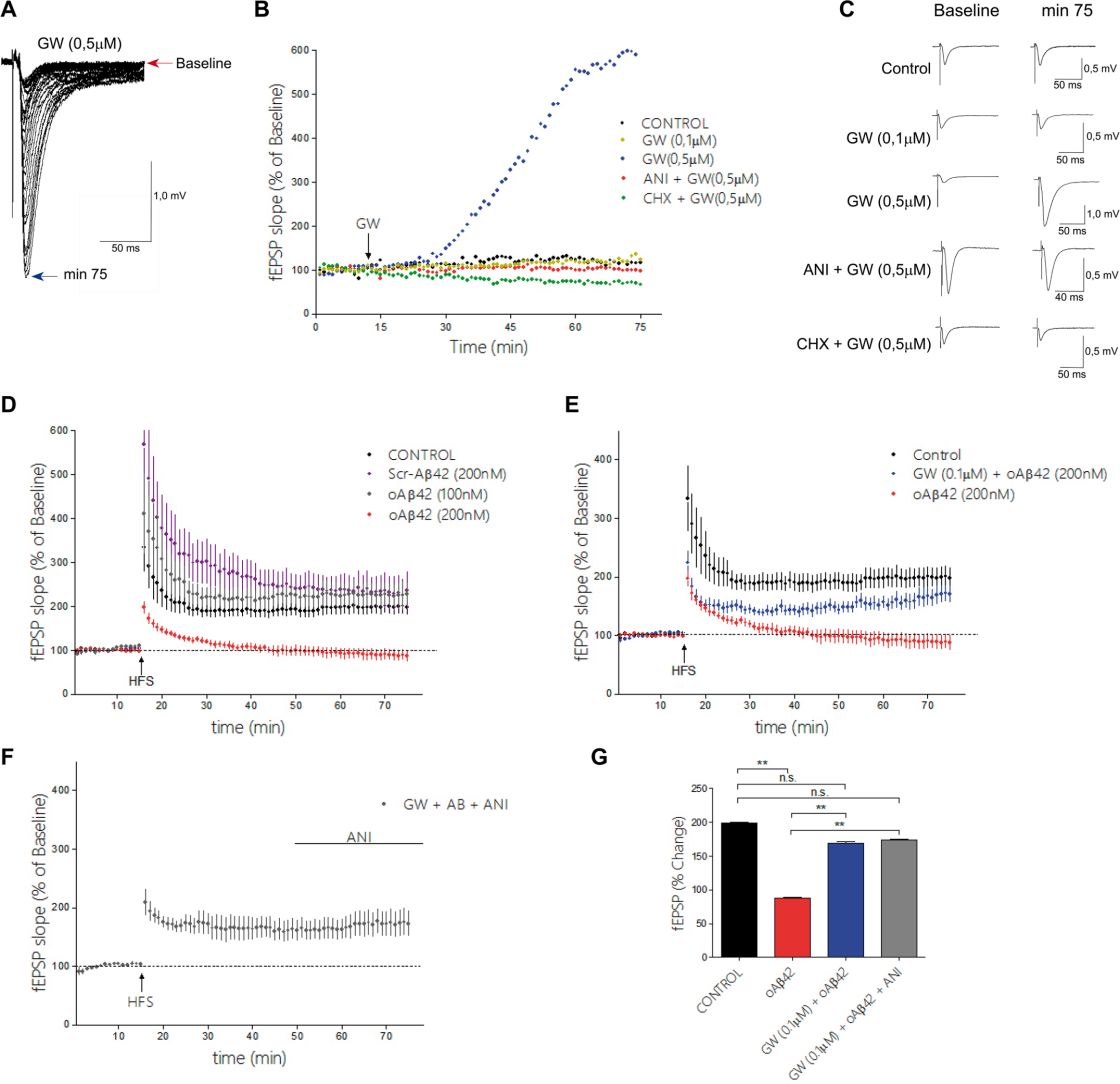
GW3965 modulates CA1 fEPSPs and prevents synaptic plasticity impairment mediated by Aβ42-oligomers. **(A)** Representative examples of evoked fEPSPs changes upon 0,5 μM GW3965 application (Blue dots in B), red arrow before, blue arrow identify fEPSP after 75 min of drug application. **(B)** Shown are the time-courses of field CA1 fEPSPs evoked every 30 sec (y axis = changes in fEPSP slope over time) after the following treatments: 1) GW3965 (0.1 μM) [GW 0.1 μM—yellow dots]. 2) GW3965 (0.5 μM) [GW 0.5 μM, blue dots] 3) Slices incubated for 60 min with 25 μM anisomycin (ANI) and treated with GW3965 (0.5 μM) [GW+ANI—red dots] 4) Slices incubated for 60 min with 300 μM Cicloheximide (CHX) and treated with 0.5 μM GW3965 [GW+CHX—green dots] and 5) Vehicle treated slices [control—black dots]; n = 4 per group, 3–6 months of age. In all cases a 15 min baseline fEPSP was obtained before pharmacological manipulations and fEPSP slopes were normalized to the first response. **(C)** Representative traces from B at baseline and 75 min after each treatment, as indicated. **(D)** Long term potentiation (LTP) was elicited by one train of 100 stimuli at 100 Hz (arrow), fEPSPs were evoked every 30 sec. Shown are normalized fEPSP slope values, the data is presented as mean +/-SEM. Slices were incubated with oligomeric (oAβ42), scrambled (sAβ) or vehicle (control) during 40 min before recording and LTP was induced after 15 min of baseline. [Control-vehicle treated, n = 7- black dots; oAβ42 100nM, n = 4—gray dots; oAβ42 200 nM, n = 5—red dots and sAβ 200 nM, n = 6—purple dots]. **(E)** Shown are time-courses of fEPSPs responses after high frequency stimulation (HFS) as in D, in slices incubated with GW3965 0.1 μM for 60 min and 200 nM oAβ42 for 40 min [GW+ oAβ42, n = 5—blue dots]. For comparative purposes traces of slices incubated with 200 nM oAβ42 [red dots] and controls [black dots] from D were plotted. **(F)** Shown are traces of fEPSP responses in slices incubated with GW3965 0.1 μM for 60 min and 200 nM oAβ42 for 40 min. After 30 min of HFS, 25 μM anisomycin (ANI) was added to the bath [GW+ oAβ42+ANI], as indicated by line. **(G)** Bar graphs and two way ANOVAs analysis of fEPSPs changes from baseline at the 50–60 min interval after HFS in control, oAβ42 200 nM, [GW 0.1 μM + 200 nM oAβ42] and [GW + oAβ42 200nM + ANI 25 μM], ** denotes p<0.01.

At the functional level, AD has been proposed to be characterized by early synaptic abnormalities [37]. AD mouse models display changes in synaptic structure, function and plasticity at a young age, [38,39]. It has also been hypothesized that synaptic dysfunction is a major cause of the cognitive deficits observed in AD [27,29,40]. Because GW3965 improved cognitions in an hippocampus-dependent paradigm such as MWM, we tested whether the drug had any effect on hippocampus synaptic transmission. Acute application of 0.5 μM GW3965, produced a gradual potentiation of single fEPSPs evoked from Schaeffer collateral to CA1 neurons every 30 sec (Fig 9A), which was prevented by the protein synthesis inhibitors anisomycin or cyclohexemide [41] (Fig 9B and 9C). Interestingly 0.1 μM GW3965 did not significantly affected fEPSP amplitude or slope (Fig 9B and 9C), while 1 μM was synaptotoxic (not shown). As it has been reported before [42] also in a dose dependent manner, bath application of oligomeric Aβ42 (oAβ42) peptides at 200 nM concentration in hippocampal brain slices of WT mice inhibited the induction of long term potentiation of the Schaeffer collateral-CA1 synapses, a proposed electrophysiological correlate to learning and memory (Fig 9D). No significant effect on LTP induction or maintenance was observed with 100 nM (oAβ42) peptides or scrambled control Aβ peptides (Fig 9D). Since for the LTP paradigm it is necessary to obtain a baseline fEPSP for 15 min (see methods for details) we used 0.1 μM GW3965 co-incubation with oAβ42, as shown in (Fig 9E and 9G), GW3965 at this concentration rescued LTP function. These experiments suggest that GW3965 at 0.1 μM independently of the acute effect on fEPSP amplitude or slope can rescue LTP. We also rule out that the slice incubation with 200 nM oAβ42 or 0.1 μM GW3965+ 200 nM oAβ42 had any effect on basal fEPSP amplitude or slope for the time of the recording (S4 File). We then asked whether the effect of GW3965 on LTP modulation by oAβ42 required protein synthesis. Since LTP induction has been shown to be protein synthesis dependent [43,44], anisomycin was used to block protein synthesis 30 min after LTP was induced, as shown in (Fig 9F). The effect of GW was not affected by this treatment. These experiments are consistent with the observations made by other groups, that LTP induction is protein synthesis dependent, but not its maintenance (for the time we studied here). The experiments also suggest that once the effect of GW3965 is established (during the incubation period) then it is independent of protein synthesis. This does not imply that protein synthesis is not required during its initial effect. [45–47].

## Discussion

LXR activation has become a promising therapeutic target as demonstrated by its ability to improve cognitive function in murine models of AD. In the present study, we addressed the molecular/functional mechanisms associated with the beneficial effects exerted by LXR treatment in the 3xTg-AD murine model of AD, and in particular those not affecting the amyloid load.

First, we found that long-term treatment (3 months) with an LXR agonist improved performance on learning, retention and transference tasks in 3xTg-AD as compared with untreated 3xTg-AD mice. Previous studies using the same LXR agonist treatment in the Tg2576 mice model of AD have demonstrated restoration of cognitive (memory) impairment by using a contextual fear-conditioning paradigm [18].

Second, we observed increased expression levels of ApoE and ABCA1 proteins, which are direct target genes regulated by LXRs, and recently reported to be expressed *in vitro* in primary culture of cortical neurons [48]. A previous study reported a 4-fold increase of ABCA1 levels in cerebral cortex and a 6-fold increase in hippocampus in APP/PS1 mice treated for 8 weeks as compared to untreated APP/PS1. Such increases were necessary for the cognitive improvement mediated by LXR activation [19]. In the present study, we observed a similar trend; a 3-fold increase in ABCA1 expression in the cerebral cortex and hippocampus in treated 3xTg-AD compared with untreated 3xTg-AD. Interestingly, the increase in ABCA1 expression in treated WT compared with untreated WT, was 6-fold in cerebral cortex and 3-fold in the hippocampus without causing a difference in cognition performance tasks between such groups. We also observed that ApoE expression was 2-fold higher in the hippocampus when comparing treated and untreated 3xTg-AD mice. However, no significant changes were observed in cerebral cortex. Similar data have been reported previously [18,19]. A significant difference in the present study is that we observed these changes in mice expressing both Aβ and tau-related pathologies, while the aforementioned studies used amyloidogenic AD mouse models. Of note, there is clear evidence of interaction of the two pathologies at different levels [49–53].

Functional studies of ApoE have demonstrated that it contributes to acceleration of the clearance of Aβ mediated by pharmacological LXR activation [18,23]. However, we showed that the increased ApoE overexpression in the brain was not accompanied by changes in the soluble amyloid beta (1–40 and 1–42), amyloid load or phosphorylated-tau levels following LXR agonist treatment in the present paradigm. Therefore, the beneficial effects of the LXR treatment herein may be independent of a detectable reduction in the histopathological hallmarks of AD.

We report differences in the expression levels of ApoE between treated and untreated WT mice using WB analysis, but this trend was not observed by using immunofluorescence analysis. We believe that such differences may be explained by the differences in the type of samples used for each technique. Protein extracts used for WB (RIPA) were prepared using samples that contained blood and other cerebral fluids, while for the immunofluorescence the samples did not contain cerebral fluids and the staining was specific for certain group of cells. In addition, LXR activation has been described to increase the reverse cholesterol transport, which subsequently induced an up-regulation of genes such as ApoE and ABCA1, promoting the cholesterol output into the cerebral fluid [54,55].

The distribution pattern of increased expression of ApoE by LXR agonist GW3065 in the hippocampus and entorhinal cortex of 3xTg-AD-treated mice, indicates that the LXR agonist acts in a cell- and region-specific manner in 3xTg-AD mice as compared to WT mice. This hippocampal subregional selectivity coincides closely with the anatomical pattern of AD pathology [1,3]. This pattern of distribution may be the result of changes occurring along the development of the pathological phenotype, including microglial remodeling in the DG or associated with acute astrogliosis [56], as it is also observed in the present paper. Consistent with previous reports showing an anti-inflammatory activity of LXR agonists [17], the present results also indicate reduction of astrogliosis in treated 3xTg-AD mice.

The cellular distribution of ApoE reported here differs from previous reports. It has been shown that in APP23 mice treated for 7 weeks with the LXR agonist TO901317, ApoE localized mainly in astrocytes and this was associated with reduction of the insoluble and soluble Aβ levels (80% and 40% respectively) [21]. We did not find increased levels of ApoE in astrocytes and only a no-significant reduction in the amyloid load was found. Furthermore, in treated 3xTg-AD, we found that cells that overexpressed ApoE in the GCL of DG did not colocalize with GFAP but they did so with NeuN suggesting that neurons are the main source of ApoE in the present analysis. Increased ApoE expression may be associated with injury of neurons, as previously described [35], which in our model may be caused by abnormal tau [49,57]. However, our experimental model does not allow us to conclude that ApoE is mainly generated by neurons. It has been reported LXR-mediated transcriptional activity in neurons which express ApoE and ABCA1 [48]. The other possibility is that ApoE is accumulated in mature neurons by lipoprotein receptor-mediated endocytosis, [21] The source of ApoE and the changes in localized expression of ABCA1 after treatment deserve a more detailed analysis.

Third, we found increased NSC and proliferating cells in the SGZ of DG in treated mice compared with untreated 3xTg-AD mice. The SGZ has been widely described as the main germinal niche for NSC and the place where neurogenesis initiates in the hippocampus [58,59]. Dysfunction of neurogenesis in the DG area has been associated with AD in different transgenic animal models expressing mutant presenilin-1, mutant APP and in the 3xTg-AD [60–66] The evidence indicates that adult neurogenesis in the SGZ begins with a population of astrocytes called radial astrocytes that express ApoE, which is required for increasing neurogenesis [67,68]. Importantly, we observed colocalization between ApoE and nestin in the SGZ of the hippocampus, in particular within proliferating cells. The relevance of ApoE for the NSC physiology is poorly understood. In ApoE-null mice, it has been reported that ApoE is required for the maintenance of the progenitor pool, for neurogenesis in the adult hippocampus as well as for the maturation of neuroblasts into neurons [69].

This result agrees with reports that showed that LXR promotes neurogenesis *in vitro* from human embryonic stem cells and *in vivo* in the ventral midbrain during development [15,70,71]. The recovery of the number of NSCs may indicate a normal restoration of the hippocampus and the presence of NSC in DG may be generating neuroprotective mediators that contribute to recovery of dysfunctional neurons [72,73].

Finally, we showed that GW3965 enhances synaptic transmission in a protein synthesis dependent manner. Different reports have showed a reduction of transduction of specific proteins in AD [74–76]. The fact that ribosomal activity is required to observe the effect of GW3965 on synaptic function, suggests that LXR regulates rapidly protein synthesis related to synaptic function. Since LXR regulates neuronal differentiation [71] this suggests that it may be involved in the transcription of genes implicated in synaptic mechanisms. LXR also shows a non-nuclear function through a direct interaction with ERα in endothelial cells, where it mediates Akt phosphorylation [77]. Akt is the main regulator of MTOR, a major pathway that controls transduction in several cell types, but this type of regulation in neurons remains unclear. We also found that pretreatment of hippocampal slices with GW3965 prevents the LTP inhibition caused by oAβ42. Since GW3965 does not exert LTP recovery in a protein synthesis dependent fashion, that suggests that LXR counteracts against the deleterious effect of oAβ42 before and/or during the LTP induction [41].

Altogether, these results suggest that several molecular and cellular changes are taking place under LXR agonist treatment. These changes could potentially explain the cognitive recovery in 3xTg-AD mice. It remains to be clarified, if ApoE is the leading actor in the regenerative and remodeling process observed in the hippocampus. It is also possible that LXR agonists modulate currently unknown genes which directly or indirectly counteract the harmful effects of Aβ and/or tau pathologies; however future experiments must be developed to validate it. Furthermore, previously has been described that interaction of ApoE with its receptors may affect diverse signaling pathways, modulating synaptic plasticity, dendritic spine development, neurite growth, lamination of neuronal layers in the DG, regulation of migration and cellular transport [78,79]

Our findings also indicate that LXR activation in murine models of AD promotes anti-inflammatory response and recovery of synaptic function through the modulation of synaptic-related protein synthesis counteracting Aβ effect, especially in the hippocampus. Under a long term treatment with GW3965 astrogliosis is reduced and neurogenesis function is recovered, both of which may add complementary mechanisms for the improvement in synaptic function at the cellular level and memory at the behavioral level in GW3965 treated AD mice. In conclusion, we described plausible cellular, molecular and functional mechanisms by which LXRs agonist GW3965 has therapeutic potential on the recovery of the cognitive impairment observed in the 3xTg-AD murine model.

## Supporting Information

S1 File No detectable reduction in amyloid beta. Amyloid plaque deposition in brains of treated and untreated mice groups were evaluated by Thio-S staining. **(Figure A and Figure B)** Representative micrographs of brain stained sections. Data were expressed as mean ± S.E.M. Statistical analysis was done by one way ANOVA followed by Tukey's multiple comparison test. n = 4 per group.
(PDF)

S2 File ApoE immunoreactivity increases in GCL but not in the ML of DG in GW3965-treated 3xTg-AD. **(Figure A)** Representative micrographs of GFAP (Red) and ApoE (Green) immunohistochemistry using confocal microscopy showing DG subregion, **(Figure B)** RFI of ApoE in the ML of DG of the hippocampus. **(Figure C)** RFI of ApoE in the GCL of DG of the hippocampus. Data were expressed as mean ± S.E.M. Statistical analysis was performed by one-way ANOVA followed by Bonferroni post test. Differences against untreated WT: *, P>0.05. Differences against untreated 3xTg-AD, #, P>0.05 Females n = 4 per group.
(PDF)

S3 File LXR agonist increases ApoE in NeuN positive cell without any increase of the neuronal cells number. **(Figure A, Figure B, Figure C and Figure D)** Representative micrographs of NeuN (Red), ApoE (Green) immunofluorescence and Hoechst (Blue) using confocal microscopy on x20 of magnification in DG, CA3 and CA1 of the hippocampus. **(Figure E)** Representative micrographs of NeuN immunofluorescence in DG of the hippocampus, the magnification showed the region used to the thickness analyze. **(Figure F)** Comparative analysis of thickness of NeuN positive immunoreactivity in the DG of the Hippocampus, Data were expressed as mean ± S.E.M. Statistical analysis was performed by one-way ANOVA followed by Bonferroni post test.*: P>0.05.**: P>0.01. n = 4 per 3xTg AD group.
(PDF)

S4 File High concentration of neither oAβ42 or oAβ42 plus GW3965 affect single evokedCA1 fEPSPs. fEPSPs slope over time after the following treatments: 1) 200 nM oAβ42 [n = 4—black dots] for 40 min and 2) 0.1 μM GW3965 (1 hour incubation before recording) and 40 min 200 nM oAβ42 [n = 4—red dots].
(PDF)

S5 File GW3965 treatment did not reduce amyloid plaque area in Subiculum, CA1 of the hippocampus and Entorhinal cortex in 3xTg-AD mice. Amyloid plaque deposition in brains of treated and untreated mice groups were evaluated byimmunohistochemistry with antibody anti-Aβ (6E10). **(Figure A)** Representative pictograms of Aβ immunohistochemistry from regions of interest; Subiculum, CA1 of the hippocampus and Entorhinal cortex. **(Figure B)** Quantitative analysis of the data presented in A form Subiculum. **(Figure C)** CA1 of the hippocampus and **(Figure D)** Entorhinal cortex (Ent Cx). Not significant changes were found in Aβ plaque area after GW3965 treatment in 3xTg-AD mice as compared with untreated 3xTg-AD mice. Data were expressed as mean ± S.E.M. Statistical analysis was done by one way ANOVA followed by Tukey's multiple comparison test. Females, n = 4 per group.
(PDF)
