# Supplementary material for: Role of Liver X Receptor in AD Pathophysiology
Source: PLoS One. 2015 Dec 31;10(12):e0145467. doi: 10.1371/journal.pone.0145467 (PMC4697813; doi:10.1371/journal.pone.0145467)
Supplement: S1 File — Amyloid plaque deposition in brains of treated and untreated mice groups were evaluated by Thio-S staining. (Figure A and Figure B) Representative micrographs of brain stained sections. Data were expressed as mean ± S.E.M. Statistical analysis was done by one way ANOVA followed by Tukey's multiple comparison test. n = 4 per group. (PDF) [file pone.0145467.s001.pdf]

Figure S1.

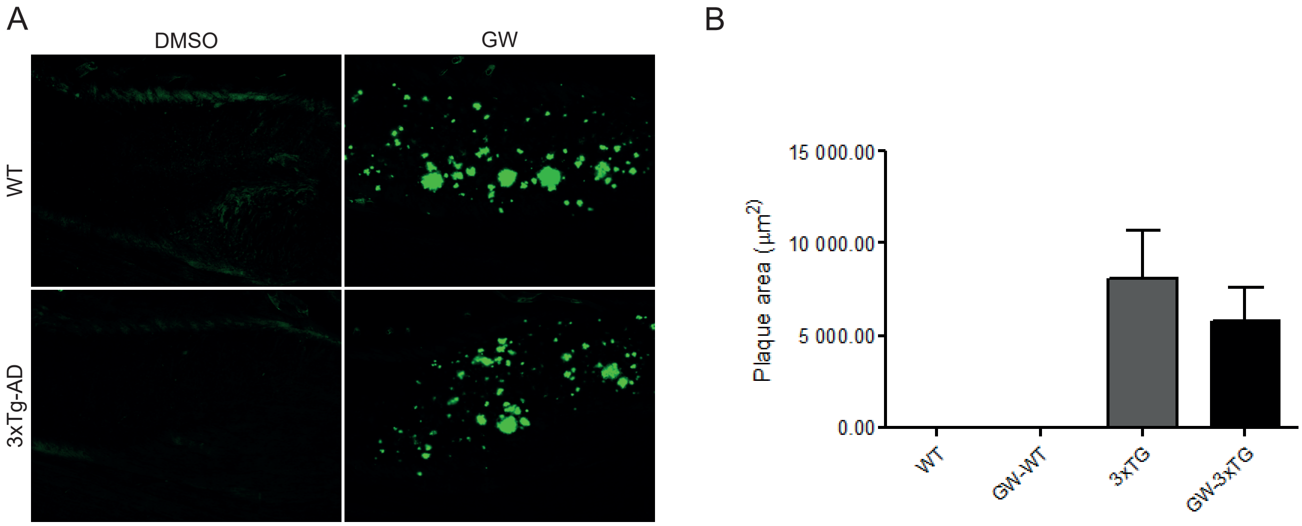

Figure S1. No detectable reduction in amyloid beta. Amyloid plaque deposition in brains of treated and untreated mice groups were evaluated by Thio-S staining. **(A-B)** Representative micrographs of brain stained sections. Data were expressed as mean ± S.E.M. Statistical analysis was done by one way ANOVA followed by Tukey's multiple comparison test. n=4 per group.
