# Supplementary material for: Role of Liver X Receptor in AD Pathophysiology
Source: PLoS One. 2015 Dec 31;10(12):e0145467. doi: 10.1371/journal.pone.0145467 (PMC4697813; doi:10.1371/journal.pone.0145467)
Supplement: S2 File — (Figure A) Representative micrographs of GFAP (Red) and ApoE (Green) immunohistochemistry using confocal microscopy showing DG subregion, (Figure B) RFI of ApoE in the ML of DG of the hippocampus. (Figure C) RFI of ApoE in the GCL of DG of the hippocampus. Data were expressed as mean ± S.E.M. Statistical analysis was performed by one-way ANOVA followed by Bonferroni post test. Differences against untreated WT: *, P>0.05. Differences against untreated 3xTg-AD, #, P>0.05 Females n = 4 per group. (PDF) [file pone.0145467.s002.pdf]

Figure S2.

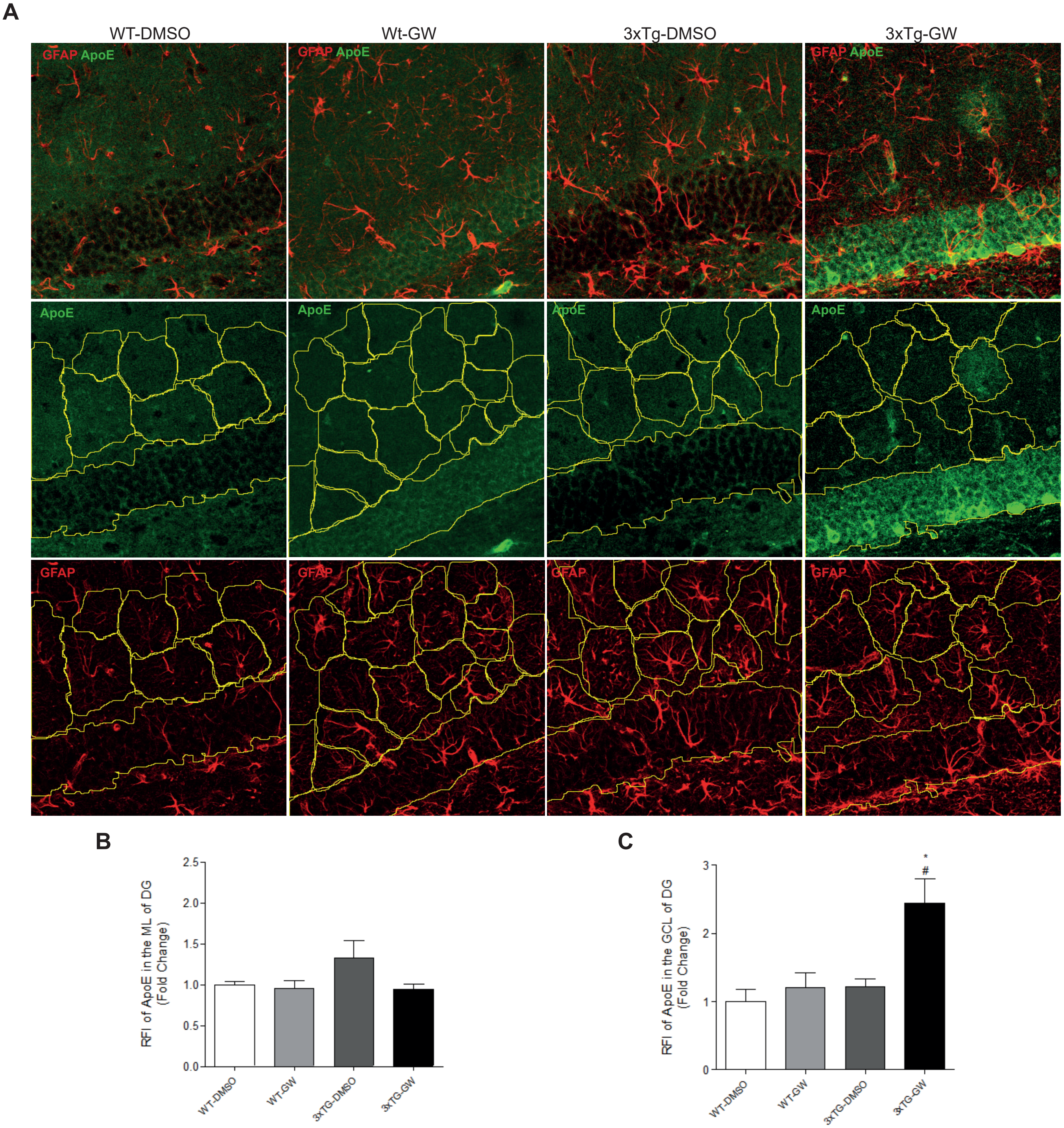

Figure S2. ApoE immunoreactivity increases in GCL but not in the ML of DG in GW3965-treated 3xTg-AD. (A) Representative micrographs of GFAP (Red) and ApoE (Green) immunohistochemistry using confocal microscopy showing DG subregion, (B) RFI of ApoE in the ML of DG of the hippocampus. (C) RFI of ApoE in the GCL of DG of the hippocampus. Data were expressed as mean ± S.E.M. Statistical analysis was performed by one-way ANOVA followed by Bonferroni post test. Differences against untreated WT: *, P>0.05. Differences against untreated 3xTg-AD, #, P>0.05 Females n=4 per 3xTg AD group.
