# Supplementary material for: Role of Liver X Receptor in AD Pathophysiology
Source: PLoS One. 2015 Dec 31;10(12):e0145467. doi: 10.1371/journal.pone.0145467 (PMC4697813; doi:10.1371/journal.pone.0145467)
Supplement: S3 File — (Figure A, Figure B, Figure C and Figure D) Representative micrographs of NeuN (Red), ApoE (Green) immunofluorescence and Hoechst (Blue) using confocal microscopy on x20 of magnification in DG, CA3 and CA1 of the hippocampus. (Figure E) Representative micrographs of NeuN immunofluorescence in DG of the hippocampus, the magnification showed the region used to the thickness analyze. (Figure F) Comparative analysis of thickness of NeuN positive immunoreactivity in the DG of the Hippocampus, Data were expressed as mean ± S.E.M. Statistical analysis was performed by one-way ANOVA followed by Bonferroni post test.*: P>0.05.**: P>0.01. n = 4 per 3xTg AD group. (PDF) [file pone.0145467.s003.pdf]

Figure S3

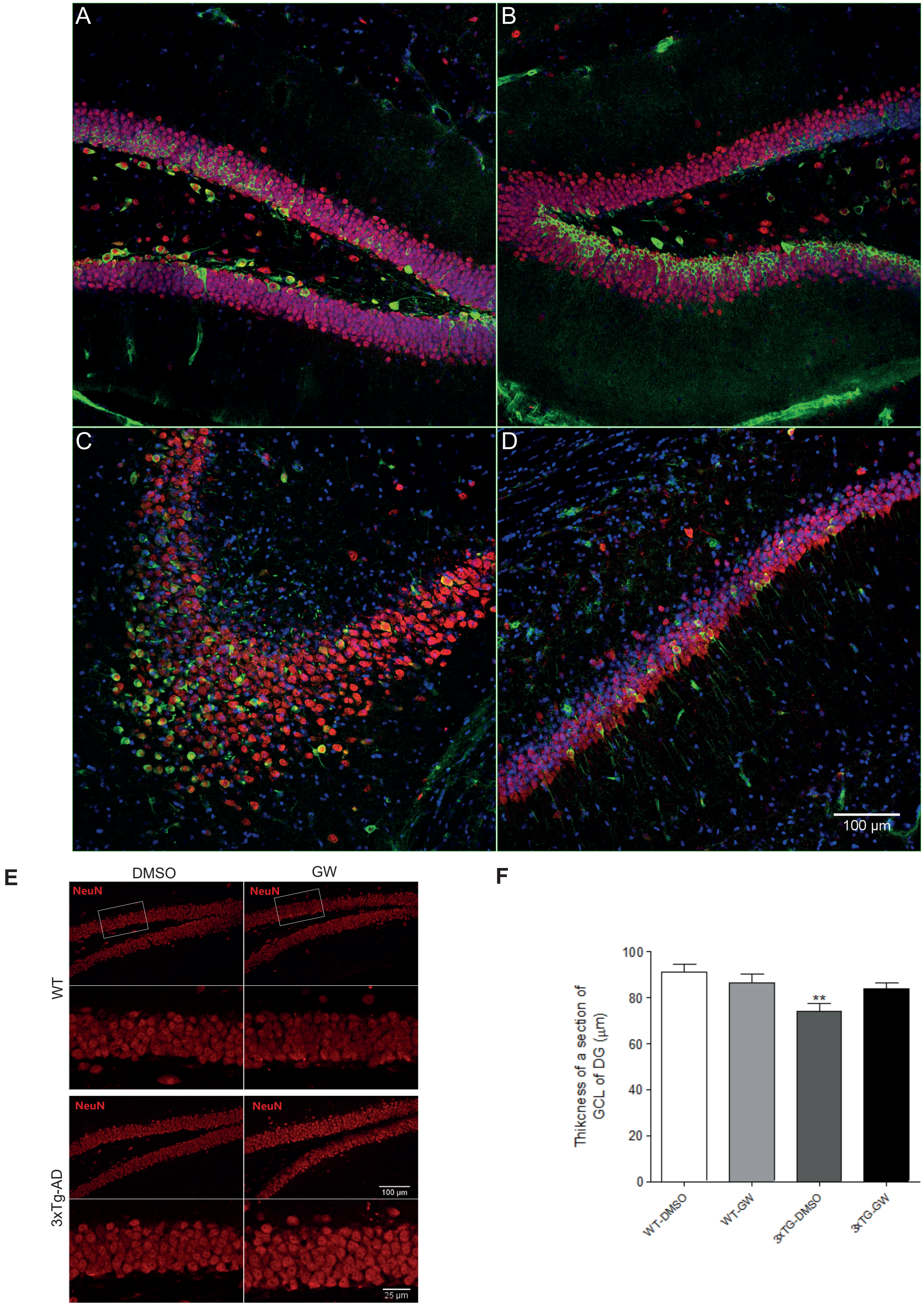

A
B
C
D
100 μm
E
DMSO
GW
NeuN
WT
3xTg-AD
100 μm
25 μm
F
Thikcness of a section of GCL of DG (μm)
100
80
60
40
20
0
**
WT-DMSO
WT-GW
3xTG-DMSO
3xTG-GW

Figure S3. LXR agonist increases ApoE in NeuN positive cellwithout any increase of the neuronal cells number. (A-D) Representative micrographs of NeuN(Red), ApoE (Green) immunofluorescence and Hoechst (Blue) using confocal microscopy on x20 of magnification in DG, CA3 and CA1 of the hippocampus. **(E)** Representative micrographs of NeuN immunofluorescence in DG of the hippocampus, the magnification showed the region used to the thickness analyze. **(F)** Comparative analysis of thickness of NeuN positive immunoreactivity in the DG of the Hippocampus, Data were expressed as mean ± S.E.M. Statistical analysis was performed by one-way ANOVA followed by Bonferroni post test.*: P>0.05.**: P>0.01. n=4 per 3xTg AD group.
