# Supplementary material for: Role of Liver X Receptor in AD Pathophysiology
Source: PLoS One. 2015 Dec 31;10(12):e0145467. doi: 10.1371/journal.pone.0145467 (PMC4697813; doi:10.1371/journal.pone.0145467)
Supplement: S4 File — fEPSPs slope over time after the following treatments: 1) 200 nM oAβ42 [n = 4—black dots] for 40 min and 2) 0.1 μM GW3965 (1 hour incubation before recording) and 40 min 200 nM oAβ42 [n = 4—red dots]. (PDF) [file pone.0145467.s004.pdf]

Figure S4.

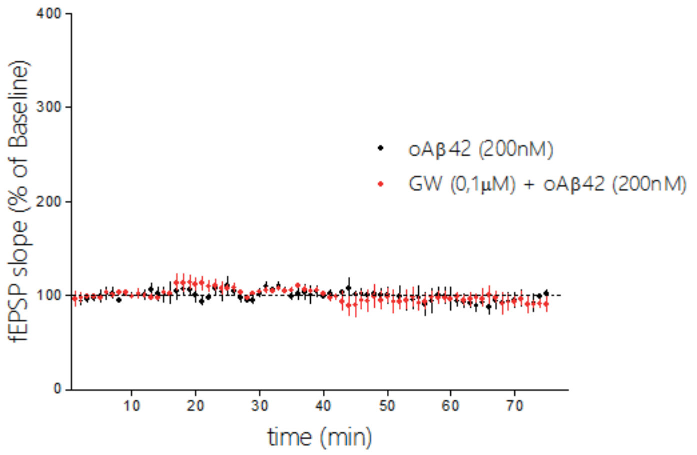

Figure S4. High concentration of neither oAβ42 or oAβ42 plus GW3965 affect single evoked CA1 fEPSPs. fEPSPs slope over time after the following treatments: 1) 200 nM oAβ42 [n=4--black dots] for 40 min and 2) 0.1 μM GW3965 (1 hour incubation before recording) and 40 min 200 nM oAβ42 [n=4—red dots].
