# Supplementary material for: Role of Liver X Receptor in AD Pathophysiology
Source: PLoS One. 2015 Dec 31;10(12):e0145467. doi: 10.1371/journal.pone.0145467 (PMC4697813; doi:10.1371/journal.pone.0145467)
Supplement: S5 File — Amyloid plaque deposition in brains of treated and untreated mice groups were evaluated byimmunohistochemistry with antibody anti-Aβ (6E10). (Figure A) Representative pictograms of Aβ immunohistochemistry from regions of interest; Subiculum, CA1 of the hippocampus and Entorhinal cortex. (Figure B) Quantitative analysis of the data presented in A form Subiculum. (Figure C) CA1 of the hippocampus and (Figure D) Entorhinal cortex (Ent Cx). Not significant changes were found in Aβ plaque area after GW3965 treatment in 3xTg-AD mice as compared with untreated 3xTg-AD mice. Data were expressed as mean ± S.E.M. Statistical analysis was done by one way ANOVA followed by Tukey's multiple comparison test. Females, n = 4 per group. (PDF) [file pone.0145467.s005.pdf]

Figure S5.

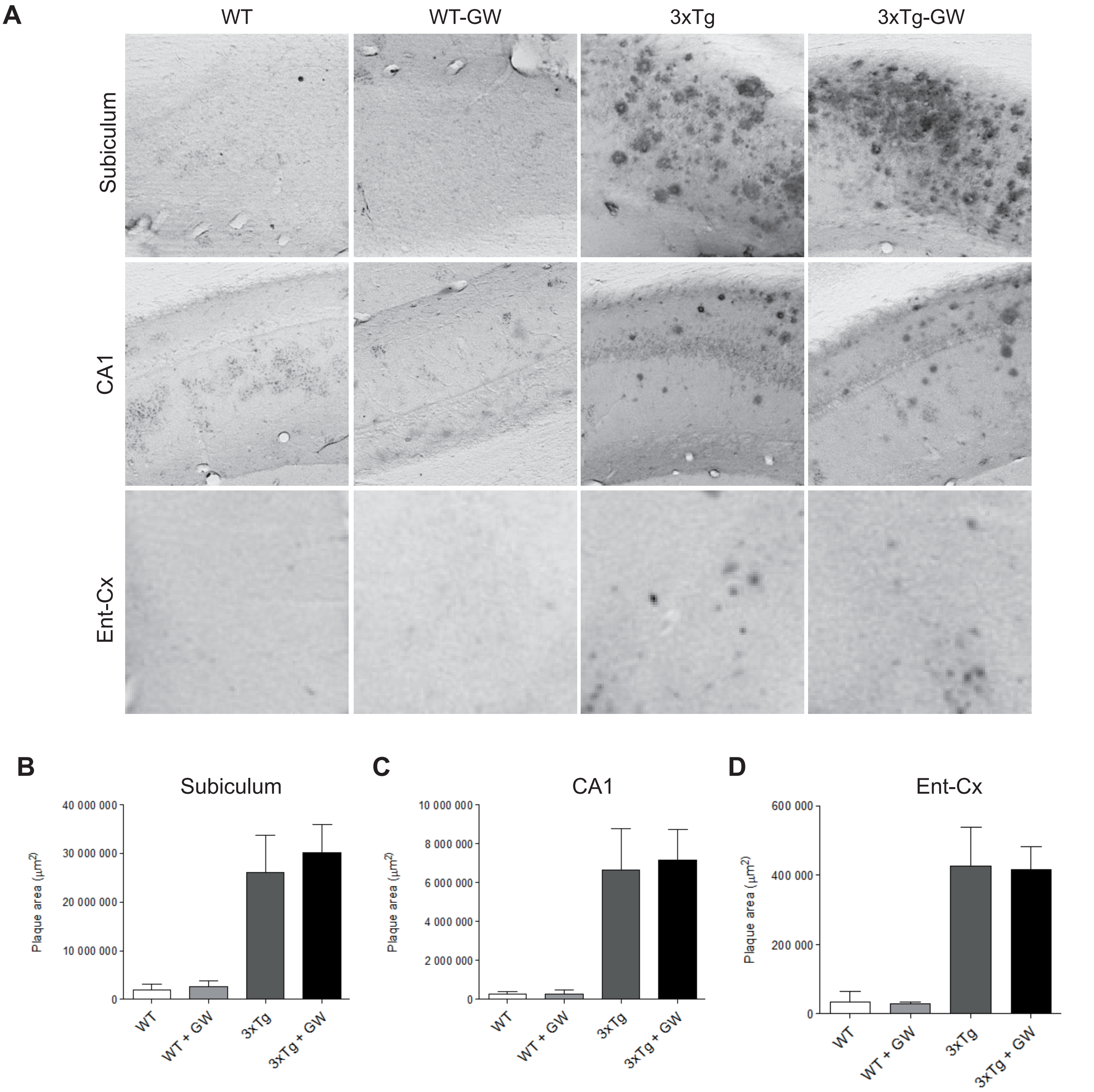

Figure S5. GW3965 treatment did not reduce amyloid plaque area in Subiculum, CA1of the hippocampus and Entorhinal cortex in 3xTg-AD mice. Amyloid plaque deposition in brains of treated and untreated mice groups were evaluated byimmunohistochemistry with antibody anti-Aβ (6E10). (A) Representative pictograms of Aβ immunohistochemistry from regions of interest; Subiculum, CA1 of the hippocampus and Entorhinal cortex. (B) Quantitative analysis of the data presented in A form Subiculum. (C) CA1 of the hippocampus and (D) Entorhinal cortex (Ent Cx). Not significant changes were found in Aβ plaque area after GW3965 treatment in 3xTg-AD mice as compared with untreated 3xTg-AD mice. Data were expressed as mean ± S.E.M. Statistical analysis was done by one way ANOVA followed by Tukey's multiple comparison test. Females, n=4 per group.
